# Supplementary material for: Reproducibility Crossroads: Impact of Statistical Choices on Proteomics Functional Enrichment
Source: Int J Mol Sci. 2025 Sep 21;26(18):9232. doi: 10.3390/ijms26189232 (PMC12471179; doi:10.3390/ijms26189232)
Supplement: Supplementary file 1 [file ijms-26-09232-s001.zip › SupplementaryFiles/Captions_Supplementary.pdf]

Figure S1, Density plots of -log10(adjusted p-value) distributions for different hypothesis testing methods across five quantitative proteomics Works and Principal Component Analysis (PCA) plots.

Figure S2, Distributions of Protein Intensities and Statistical Metrics Across Workflows (W1-W5). This figure displays various distributions derived from quantitative proteomics data for each of the five comparative workflows after hypothesis testing (W1 to W5). The left panels illustrate the overall distribution of protein intensities (likely log-transformed LFQ intensities) and the distribution of adjusted p-values. The right panels provide more detailed histograms showing the distribution of log2 fold changes s for proteins identified by each statistical method (t-Student, t-Welch, limma, DEqMS, MSstats, Bayesian). The colored bars/lines within each panel differentiate between conditions or between individual replicates, providing insights into data quality, variability, and the performance of different statistical approaches across the analyzed datasets.

Figure S3, Comparison of overlaps in the significant proteins discarded after applying the filters according to the results of the Bayesian analysis and applying the |log2FC| of 1 in the Work1.

Figure S4, Correlation and Similarity Distributions and Pairwise Methodological Heatmaps for Work 1. This figure presents various analyses of correlation and similarity metrics for Work 1, across different methodological comparisons and directions of regulation. All panels in this figure pertain exclusively to Work 1. Upper part: Correlation Coefficient Distributions. Boxplots illustrating the distributions of Pearson and Spearman correlation coefficients. These panels represent data for the 'up' category, aggregating across all ontologies. (Note: Similar boxplots for the 'down' category are also included in the full figure). The Kruskal-Wallis test p-values for overall group differences are indicated, along with pairwise statistical significances denoted by asterisks. Lower part: Ontology-Specific Pairwise Methodological Heatmaps. These heatmaps display the similarity between methodological combinations for 3 specific ontologies: GO Biological Process, GO Molecular Function, and KEGG. For each ontology, similarity is represented using Euclidean similarity, Jaccard index, Pearson correlation, and Spearman correlation. The heatmaps are organized in four columns forming two "blocks" from left to right: The left block, comprising two columns, represents the 'up' category. The right block, also comprising two columns, represents the 'down' category. Within each heatmap, the color intensity indicates the similarity value, with cooler colors (darker blue) representing higher similarity and warmer colors (red) indicating lower similarity between the methodological combinations on the axes.

Figure S5, Global distribution and comparison of Pearson and Spearman indices from meta-analysis. A and C) Histogram showing the distribution of the raw Pearson and Spearman indices values, respectively across all collected data (left), histogram of the arcsin(sqrt)-transformed values, illustrating how the transformation affects the data distribution (right) and boxplots representing the arcsin(sqrt)-transformed Euclidean similarity and Jaccard Index values, categorized by four comparison types: "Intra-CBR_Fixed_HTM" (concordance among CBRs with fixed HTM), "Intra-HTM_Bayes_CBR" (concordance among Hypothesis Testing Methods with Bayesian-based Criteria for Biological Relevance), "Inter-HTM_Inter-CBR" (concordance when both HTM and CBR vary), "Intra-HTM_FC_CBR" (concordance among HTMs with FC-based CBR) (down). Asterisks denote significance from post-hoc Wilcoxon rank-sum tests (ns: not significant,

* p < 0.05, ** p < 0.01, *** p < 0.001, **** p < 0.0001). B and D) Sensitivity Analysis of Kruskal-Wallis Test for Pearson and Spearman indices. Bar plot showing the Kruskal-Wallis p-value for the global comparison when sequentially excluding one 'Work' (dataset) at a time from the meta-analysis. The red dashed line at p=0.05 serves as a significance threshold.

Table S1, MaxQuant (v2.1.4.0) Search and Processing Parameters. Parameters listed below were consistently applied across all five proteomic works (Works 1-5) analyzed in this study, with the only variation being the specific FASTA database selected for each respective organism.

Table S2, Summary of interactions between the different methods implemented, separated by overrepresented and underrepresented.

Table S3, Summary of interactions between the different methods implemented, separated by overrepresented and underrepresented.
