# Supplementary material for: Reproducibility Crossroads: Impact of Statistical Choices on Proteomics Functional Enrichment
Source: Int J Mol Sci. 2025 Sep 21;26(18):9232. doi: 10.3390/ijms26189232 (PMC12471179; doi:10.3390/ijms26189232)
Supplement: Supplementary file 1 [file ijms-26-09232-s001.zip › SupplementaryFiles/Table S1.pdf]

Table S1. MaxQuant (v2.1.4.0) Search and Processing Parameters. Parameters listed below were consistently applied across all five proteomic works (Works 1-5) analyzed in this study, with the only variation being the specific FASTA database selected for each respective organism.

Parameter	*Value*	*Description*
Software Version	2.1.4.0	MaxQuant software version used for data processing.
User name	Alfonso	
Machine name	MSI	
Contaminant Inclusion	TRUE	Specifies if common contaminants (e.g., from contaminants.fasta) were considered during search.
False Discovery Rate (FDR)		
PSM FDR	0.01	Peptide-Spectrum Match FDR (1%).
PSM FDR Crosslink	0.01	
Protein FDR	0.01	Protein FDR (1%).
Site FDR	0.01	Phospho (or other site) FDR.
Use Normalized Ratios For Occupancy	TRUE	
Peptide Parameters		
Min. peptide Length	7	Minimum peptide length required for identification.
Min. score for unmodified peptides	0	Minimum Andromeda score for unmodified peptides.
Min. score for modified peptides	40	Minimum Andromeda score for modified peptides.
Min. delta score for unmodified peptides	0	Minimum difference between best and second-best score for unmodified peptides.
Min. delta score for modified peptides	6	Minimum difference between best and second-best score for modified peptides.
Protein Quantification		
Min. unique peptides	0	Minimum number of unique peptides for protein quantification.
Min. razor peptides	1	Minimum number of razor peptides for protein quantification.
Min. peptides	1	Minimum total peptides for protein quantification.
Use only unmodified peptides and modifications included in protein quantification	TRUE	Controls which peptides are used for quantification.
Modifications included in protein quantification	Oxidation (M); Acetyl (Protein N-term)	Variable modifications considered for protein quantification.
Peptides used for protein quantification	Razor	Strategy for using peptides for protein quantification (razor peptides are unique to a protein group).

Discard unmodified counterpart peptides	TRUE	If True, unmodified counterparts of modified peptides are discarded for quantification.
Label min. ratio count	2	
Use delta score	FALSE	
iBAQ	FALSE	Enables Intensity Based Absolute Quantification.
iBAQ log fit	FALSE	Applies logarithmic fit for iBAQ calculation.
Retention Time and Ion Mobility Alignment		
Match between runs	FALSE	Enables "Match between runs" feature to transfer identifications between runs.
"Matching time window [min]"	0.7	Retention time window for matching between runs.
"Match ion mobility window [indices]"	0.05	Ion mobility window for matching between runs.
"Alignment time window [min]"	20	Retention time window for global alignment.
"Alignment ion mobility window [indices]"	1	Ion mobility window for global alignment.
Find dependent peptides	FALSE	
FASTA Database & Decoy		
Fasta file	C:\path *.fasta	Path to the protein sequence database file.
Decoy mode	revert	Strategy for generating decoy sequences (reverted sequences).
Include contaminants	TRUE	
Fixed andromeda index folder		
Second peptides	TRUE	
LFQ Specific Parameters		
Stabilize large LFQ ratios	TRUE	Helps stabilize very large LFQ ratios.
Separate LFQ in parameter groups	FALSE	If True, LFQ is calculated separately for each parameter group.
Require MS/MS for LFQ comparisons	TRUE	Requires an MS/MS spectrum for a peptide to be used in LFQ comparison.
Mass Tolerance & Fragment Ion Parameters		
MS/MS tol. (FTMS)	20 ppm	MS/MS fragment ion mass tolerance for Fourier Transform Ion Cyclotron Resonance Mass Spectrometry (FTMS).
Top MS/MS peaks per Da interval. (FTMS)	12	Number of most intense peaks per Da interval in MS/MS spectra (FTMS).
Da interval. (FTMS)	100	Da interval for selecting top MS/MS peaks (FTMS).
MS/MS deisotoping (FTMS)	TRUE	Enables deisotoping of MS/MS spectra (FTMS).
MS/MS deisotoping tolerance (FTMS)	7	Tolerance for MS/MS deisotoping (FTMS).

MS/MS deisotoping tolerance unit (FTMS)	ppm	Unit for MS/MS deisotoping tolerance (FTMS).
MS/MS higher charges (FTMS)	TRUE	Considers higher charge states for MS/MS fragment ions (FTMS).
MS/MS water loss (FTMS)	TRUE	Considers water loss fragments in MS/MS spectra (FTMS).
MS/MS ammonia loss (FTMS)	TRUE	Considers ammonia loss fragments in MS/MS spectra (FTMS).
MS/MS dependent losses (FTMS)	TRUE	Considers dependent losses in MS/MS spectra (FTMS).
MS/MS recalibration (FTMS)	FALSE	Disables recalibration of MS/MS spectra (FTMS).
MS/MS tol. (ITMS)	0.5 Da	
Top MS/MS peaks per Da interval. (ITMS)	8	
Da interval. (ITMS)	100	
MS/MS deisotoping (ITMS)	FALSE	
MS/MS deisotoping tolerance (ITMS)	0.15	
MS/MS deisotoping tolerance unit (ITMS)	Da	
MS/MS higher charges (ITMS)	TRUE	
MS/MS water loss (ITMS)	TRUE	
MS/MS water loss (ITMS for cross link)	FALSE	
MS/MS ammonia loss (ITMS)	TRUE	
MS/MS ammonia loss (ITMS for cross link)	FALSE	
MS/MS dependent losses (ITMS)	TRUE	
MS/MS recalibration (ITMS)	FALSE	
MS/MS tol. (TOF)	25 ppm	
Top MS/MS peaks per Da interval. (TOF)	16	
Da interval. (TOF)	100	
MS/MS deisotoping (TOF)	TRUE	
MS/MS deisotoping tolerance (TOF)	0.01	
MS/MS deisotoping tolerance unit (TOF)	Da	
MS/MS higher charges (TOF)	TRUE	
MS/MS water loss (TOF)	TRUE	
MS/MS water loss (TOF for cross link)	FALSE	

MS/MS ammonia loss (TOF)	TRUE
MS/MS ammonia loss (TOF for cross link)	FALSE
MS/MS dependent losses (TOF)	TRUE
MS/MS recalibration (TOF)	FALSE
MS/MS tol. (ASTRAL)	25 ppm
Top MS/MS peaks per Da interval. (ASTRAL)	16
Da interval. (ASTRAL)	100
MS/MS deisotoping (ASTRAL)	TRUE
MS/MS deisotoping tolerance (ASTRAL)	0.01
MS/MS deisotoping tolerance unit (ASTRAL)	Da
MS/MS higher charges (ASTRAL)	TRUE
MS/MS water loss (ASTRAL)	TRUE
MS/MS water loss (ASTRAL for cross link)	FALSE
MS/MS ammonia loss (ASTRAL)	TRUE
MS/MS ammonia loss (ASTRAL for cross link)	FALSE
MS/MS dependent losses (ASTRAL)	TRUE
MS/MS recalibration (ASTRAL)	FALSE
MS/MS tol. (UNKNOWN)	20 ppm
Top MS/MS peaks per Da interval. (UNKNOWN)	12
Da interval. (UNKNOWN)	100
MS/MS deisotoping (UNKNOWN)	TRUE
MS/MS deisotoping tolerance (UNKNOWN)	7
MS/MS deisotoping tolerance unit (UNKNOWN)	ppm
MS/MS higher charges (UNKNOWN)	TRUE
MS/MS water loss (UNKNOWN)	TRUE
MS/MS water loss (UNKNOWN for cross link)	FALSE

MS/MS ammonia loss (UNKNOWN)	TRUE	
MS/MS ammonia loss (UNKNOWN for cross link)	FALSE	
MS/MS dependent losses (UNKNOWN)	TRUE	
MS/MS recalibration (UNKNOWN)	FALSE	
Site tables	Oxidation (M)Sites.txt	

Output Tables

Write msScans table	FALSE	
Write msmsScans table	TRUE	Generates the msmsScans.txt table.
Write ms3Scans table	TRUE	Generates the ms3Scans.txt table.
Write allPeptides table	FALSE	Generates the allPeptides.txt table.
Write mzRange table	TRUE	Generates the mzRange.txt table.
Write DIA fragments table	FALSE	
Write DIA fragments quant table	FALSE	
Write pasefMsmsScans table	TRUE	Generates the pasefMsmsScans.txt table (relevant for Bruker timsTOF data).
Write accumulatedMsmsScans table	TRUE	Generates the accumulatedMsmsScans.txt table.
Other Parameters		
"Max. peptide mass [Da]"	4600	Maximum peptide mass considered.
Min. peptide length for unspecific search	8	
Max. peptide length for unspecific search	25	
Razor protein FDR	TRUE	Applies FDR to razor protein groups.
Disable MD5	FALSE	
Max mods in site table	3	Maximum number of modifications displayed in the site table.
Match unidentified features	FALSE	
Epsilon score for mutations	TRUE	
Evaluate variant peptides separately	TRUE	Processes variant peptides independently.
Variation mode	None	
Site tables	Oxidation (M)Sites.txt	
