# Supplementary material for: Reproducibility Crossroads: Impact of Statistical Choices on Proteomics Functional Enrichment
Source: Int J Mol Sci. 2025 Sep 21;26(18):9232. doi: 10.3390/ijms26189232 (PMC12471179; doi:10.3390/ijms26189232)
Supplement: Supplementary file 1 [file ijms-26-09232-s001.zip › SupplementaryFiles/Table S2.pdf]

Table S2. Summary of interactions between the different methods implemented, separated by overrepresented and underrepresented.

W1

Down_Bayes

Method	*bayes_Bayes*	*deqms_Bayes*	*limma_Bayes*	*msstats_Bayes*	*tstudent_Bayes*	*twelch_Bayes*
bayes_Bayes	679	18	20	5	18	18
deqms_Bayes	18	34	34	5	33	32
limma_Bayes	20	34	38	7	33	32
msstats_Bayes	5	5	7	8	5	3
tstudent_Bayes	18	33	33	5	33	31
twelch_Bayes	18	32	32	3	31	32

Up_Bayes

Method	*bayes_Bayes*	*deqms_Bayes*	*limma_Bayes*	*msstats_Bayes*	*tstudent_Bayes*	*twelch_Bayes*
bayes_Bayes	784	68	81	81	70	68
deqms_Bayes	68	69	69	69	68	66
limma_Bayes	81	69	82	82	71	69
msstats_Bayes	81	69	82	82	71	69
tstudent_Bayes	70	68	71	71	71	69
twelch_Bayes	68	66	69	69	69	69

Down_FC

Method	*bayes_FC*	*deqms_FC*	*limma_FC*	*msstats_FC*	*tstudent_FC*	*twelch_FC*
bayes_FC	36	35	35	0	35	35
deqms_FC	35	114	114	2	111	100
limma_FC	35	114	132	2	111	100
msstats_FC	0	2	2	45	2	2
tstudent_FC	35	111	111	2	111	100
twelch_FC	35	100	100	2	100	100

Up_FC

Method	*bayes_FC*	*deqms_FC*	*limma_FC*	*msstats_FC*	*tstudent_FC*	*twelch_FC*
bayes_FC	338	204	204	195	202	186
deqms_FC	204	215	215	169	211	195
limma_FC	204	215	218	172	211	195
msstats_FC	195	169	172	1190	166	160
tstudent_FC	202	211	211	166	211	195
twelch_FC	186	195	195	160	195	195

W2

Down_Bayes

Method	*bayes_Bayes*	*deqms_Bayes*	*limma_Bayes*	*msstats_Bayes*	*tstudent_Bayes*	*twelch_Bayes*
bayes_Bayes	29	3	2	0	3	2
deqms_Bayes	3	3	2	0	3	2
limma_Bayes	2	2	2	0	2	2

Up_Bayes

Method	*bayes_Bayes*	*deqms_Bayes*	*limma_Bayes*	*msstats_Bayes*	*tstudent_Bayes*	*twelch_Bayes*
bayes_Bayes	32			0		
deqms_Bayes						
limma_Bayes						

Method	bayes_Bayes	deqms_Bayes	limma_Bayes	msstats_Bayes	tstudent_Bayes	twelch_Bayes
**msstats_Bayes**	0	0	0	8	0	0
**tstudent_Bayes**	3	3	2	0	3	2
**twelch_Bayes**	2	2	2	0	2	2

Method	bayes_Bayes	deqms_Bayes	limma_Bayes	msstats_Bayes	tstudent_Bayes	twelch_Bayes
**msstats_Bayes**	0			4		
**tstudent_Bayes**						
**twelch_Bayes**						

Down_FC

Method	***bayes_FC***	***deqms_FC***	***limma_FC***	***msstats_FC***	***tstudent_FC***	***twelch_FC***
bayes_FC	11	7	7	0	6	2
deqms_FC	7	8	8	0	6	2
limma_FC	7	8	8	0	6	2
msstats_FC	0	0	0	8	0	0
tstudent_FC	6	6	6	0	6	2
twelch_FC	2	2	2	0	2	2

Up_FC

Method	***bayes_FC***	***deqms_FC***	***limma_FC***	***msstats_FC***	***tstudent_FC***	***twelch_FC***
bayes_FC	2	2	2	0	2	
deqms_FC	2	2	2	0	2	
limma_FC	2	2	2	0	2	
msstats_FC	0	0	0	4	0	
tstudent_FC	2	2	2	0	2	
twelch_FC						

W3

Down_Bayes

Method	***bayes_Bayes***	***deqms_Bayes***	***limma_Bayes***	***msstats_Bayes***	***tstudent_FC***	***twelch_FC***
bayes_Bayes	523	4	4	0		
deqms_Bayes	4	4	4	0		
limma_Bayes	4	4	4	0		
msstats_Bayes	0	0	0	49		
tstudent_Bayes						
twelch_Bayes						

Up_Bayes

Method	***bayes_Bayes***	***deqms_Bayes***	***limma_Bayes***	***msstats_Bayes***	***tstudent_Bayes***	***twelch_Bayes***
bayes_Bayes	309	20	20	0		
deqms_Bayes	20	20	19	0		
limma_Bayes	20	19	20	0		
msstats_Bayes	0	0	0	22		
tstudent_Bayes						
twelch_Bayes						

Down_FC

Method	***bayes_FC***	***deqms_FC***	***limma_FC***	***msstats_FC***	***tstudent_FC***	***twelch_FC***
bayes_FC	6	5	5	0	-	
deqms_FC	5	6	6	0	-	
limma_FC	5	6	7	0	-	

Up_FC

Method	***bayes_FC***	***deqms_FC***	***limma_FC***	***msstats_FC***	***tstudent_FC***	***twelch_FC***
bayes_FC	37	33	33	0		
deqms_FC	33	34	34	0		
limma_FC	33	34	34	0		

Method	bayes_FC	deqms_FC	limma_FC	msstats_FC	tstudent_FC	twelch_FC
msstats_FC	0	0	0	49	-	
tstudent_FC	0	0	0	0	-	
twelch_FC						

Method	bayes_FC	deqms_FC	limma_FC	msstats_FC	tstudent_FC	twelch_FC
msstats_FC	0	0	0	22		
tstudent_FC						
twelch_FC						

W4

Down_Bayes

Method	***bayes_Bayes***	***deqms_Bayes***	***limma_Bayes***	***msstats_Bayes***	***tstudent_Bayes***	***twelch_Bayes***
bayes_Bayes	236	91	91	90	92	92
deqms_Bayes	91	99	96	92	98	97
limma_Bayes	91	96	98	92	96	96
msstats_Bayes	90	92	92	96	91	92
tstudent_Bayes	92	98	96	91	99	97
twelch_Bayes	92	97	96	92	97	99

Up_Bayes

Method	***bayes_Bayes***	***deqms_Bayes***	***limma_Bayes***	***msstats_Bayes***	***tstudent_Bayes***	***twelch_Bayes***
bayes_Bayes	309	56	57	56	57	56
deqms_Bayes	56	72	72	71	71	67
limma_Bayes	57	72	75	73	73	69
msstats_Bayes	56	71	73	73	72	68
tstudent_Bayes	57	71	73	72	73	68
twelch_Bayes	56	67	69	68	68	70

Down_FC

Method	***bayes_FC***	***deqms_FC***	***limma_FC***	***msstats_FC***	***tstudent_FC***	***twelch_FC***
bayes_FC	170	170	169	122	163	145
deqms_FC	170	209	208	141	186	162
limma_FC	169	208	228	152	186	162
msstats_FC	122	141	152	222	137	126
tstudent_FC	163	186	186	137	186	160
twelch_FC	145	162	162	126	160	162

Up_FC

Method	***bayes_FC***	***deqms_FC***	***limma_FC***	***msstats_FC***	***tstudent_FC***	***twelch_FC***
bayes_FC	176	170	169	162	144	103
deqms_FC	170	260	254	230	207	139
limma_FC	169	254	279	250	207	139
msstats_FC	162	230	250	489	198	136
tstudent_FC	144	207	207	198	207	139
twelch_FC	103	139	139	136	139	139

W5

Down_Bayes

Method	***bayes_Bayes***	***deqms_Bayes***	***limma_Bayes***	***msstats_Bayes***	***tstudent_Bayes***	***twelch_Bayes***
bayes_Bayes	112	17	16	4	8	
deqms_Bayes	17	23	21	5	10	

Up_Bayes

Method	**bayes_Bayes**	**deqms_Bayes**	**limma_Bayes**	**msstats_Bayes**	**tstudent_Bayes**	**twelch_Bayes**
bayes_Bayes	129	11	10		5	1
deqms_Bayes	11	18	17		10	5

limma_Bayes	16	21	21	5	10	
msstats_Bayes	4	5	5	5	5	
tstudent_Bayes	8	10	10	5	10	
twelch_Bayes						
			Down_FC			
Method	***bayes_FC***	***deqms_FC***	***limma_FC***	***msstats_FC***	***tstudent_FC***	***twelch_FC***
bayes_FC	88	60	52	4	8	0
deqms_FC	60	86	73	6	13	1
limma_FC	52	73	76	6	13	1
msstats_FC	4	6	6	47	6	1
tstudent_FC	8	13	13	6	13	1
twelch_FC	0	1	1	1	1	1

limma_Bayes	10	17	17		9	5
msstats_Bayes						
tstudent_Bayes	5	10	9		10	5
twelch_Bayes	1	5	5		5	5
			Up_FC			
Method	***bayes_FC***	***deqms_FC***	***limma_FC***	***msstats_FC***	***tstudent_FC***	***twelch_FC***
bayes_FC	64	38	31	0	8	2
deqms_FC	38	57	50	0	13	6
limma_FC	31	50	51	0	13	6
msstats_FC	0	0	0	27	0	0
tstudent_FC	8	13	13	0	14	6
twelch_FC	2	6	6	0	6	6
