# Supplementary material for: Reproducibility Crossroads: Impact of Statistical Choices on Proteomics Functional Enrichment
Source: Int J Mol Sci. 2025 Sep 21;26(18):9232. doi: 10.3390/ijms26189232 (PMC12471179; doi:10.3390/ijms26189232)
Supplement: Supplementary file 1 [file ijms-26-09232-s001.zip › SupplementaryFiles/Table S3.pdf]

Table S3. Summary with log2FC values and minimum and maximum Log2FC values for each implemented method, separated by overrepresented and underrepresented proteins.

Test	Mean Log2FC	Min. FC	Max. FC	Total proteins	Test	Mean Log2FC	Min. FC	Max. FC	Total proteins
**W1_Bayes_Down**	-0.524	-3.915	-0.074	679	W1_Bayes Up	1.242	0.108	4.334	784
**W1_DEqMS_Bayes_Down**	-1.808	-3.168	-1.295	34	W1_DEqMS_Bayes_up	1.773	1.219	2.947	69
**W1_Limma_Bayes_Down**	-1.806	-3.168	-1.295	38	W1_Limma_Bayes_Up	1.851	1.219	2.947	82
**W1_MSstats_Bayes_Down**	-0.957	-1.665	-0.580	8	W1_MSstats_Bayes_Up	1.668	1.052	3.500	82
**W1_TStudent_Bayes_Down**	-1.808	-3.168	-1.295	33	W1_TStudent_Bayes_Up	1.753	1.219	2.947	71
**W1_TWelch_Bayes_Down**	-1.781	-3.168	-1.295	32	W1_TWelch_Bayes_Up	1.751	1.219	2.947	69
**W2_Bayes_Down**	-0.911	-1.990	-0.270	29	W2_Bayes Up	0.625	0.228	1.560	32
**W2_DEqMS_Bayes_Down**	-1.589	-1.810	-1.427	3	W2_DEqMS_Bayes_up	-	-	-	-
**W2_Limma_Bayes_Down**	-1.670	-1.810	-1.530	2	W2_Limma_Bayes_Up	-	-	-	-
**W2_MSstats_Bayes_Down**	-3.354	-3.354	-3.354	8	W2_MSstats_Bayes_Up	-	-	-	-
**W2_TStudent_Bayes_Down**	-1.589	-1.810	-1.427	3	W2_TStudent_Bayes_Up	-	-	-	-
**W2_TWelch_Bayes_Down**	-1.670	-1.810	-1.530	2	W2_TWelch_Bayes_Up	-	-	-	
**W3_Bayes_Down**	-0.267	-2.097	-0.041	523	W3_Bayes Up	0.519	0.058	2.542	309
**W3_DEqMS_Bayes_Down**	-2.027	-2.251	-1.355	4	W3_DEqMS_Bayes_up	2.377	1.210	2.617	20
**W3_Limma_Bayes_Down**	-2.027	-2.251	-1.355	4	W3_Limma_Bayes_Up	2.377	1.210	2.617	20
**W3_MSstats_Bayes_Down**	All -inf	All -inf	All -inf	49	W3_MSstats_Bayes_Up	All Inf	All Inf	All Inf	22
**W3_TStudent_Bayes_Down**	-	-	-	-	W3_TStudent_Bayes_Up	-	-	-	-
**W3_TWelch_Bayes_Down**	-	-	-	-	W3_TWelch_Bayes_Up	-	-	-	-
**W4_Bayes_Down**	-1.492	-4.349	-0.312	236	W4_Bayes Up	1.281	0.264	7.418	309
**W4_DEqMS_Bayes_Down**	-2.442	-5.656	-1.480	99	W4_DEqMS_Bayes_up	2.881	1.361	7.989	72
**W4_Limma_Bayes_Down**	-2.434	-5.656	-1.480	98	W4_Limma_Bayes_Up	2.844	1.361	7.989	75
**W4_MSstats_Bayes_Down**	-1.986	-4.013	-1.024	96	W4_MSstats_Bayes_Up	2.408	1.213	6.357	73
**W4_TStudent_Bayes_Down**	-2.442	-5.656	-1.480	99	W4_TStudent_Bayes_Up	2.869	1.361	7.989	73
**W4_TWelch_Bayes_Down**	-2.440	-5.656	-1.480	99	W4_TWelch_Bayes_Up	2.783	1.361	7.989	70

**W5_Bayes_Down**	-1.454	-3.064	-0.605	112	W5_Bayes Up	1.098	0.265	5.184	129
**W5_DEqMS_Bayes_Down**	-2.584	-4.368	-1.855	23	W5_DEqMS_Bayes_up	2.405	1.639	5.562	18
**W5_Limma_Bayes_Down**	-2.638	-4.368	-1.855	21	W5_Limma_Bayes_Up	2.450	1.652	5.562	17
**W5_MSstats_Bayes_Down**	-2.643	-2.937	-2.076	5	W5_MSstats_Bayes_Up	-	-	-	-
**W5_TStudent_Bayes_Down**	-2.590	-4.368	-1.898	10	W5_TStudent_Bayes_Up	2.331	1.639	5.562	10
**W5_TWelch_Bayes_Down**	-	-	-	-	W5_TWelch_Bayes_Up	2.559	1.808	5.562	5
**W1_DEqMS_FC_Down**	-1.471	-4.093	-1.004	114	W1_DEqMS_FC_Up	1.536	1.021	2.947	215
**W1_Limma_FC_Down**	-1.482	-4.093	-1.004	132	W1_Limma_FC_Up	1.531	1.019	2.947	218
**W1_MSstats_FC_Down**	-1.393	-1.665	-1.120	45	W1_MSstats_FC_U	1.399	1.001	3.500	1190
**W1_TStudent_FC_Down**	-1.480	-4.093	-1.004	111	W1_TStudent_FC_Up	1.538	1.021	2.947	211
**W1_TWelch_FC_Down**	-1.498	-4.093	-1.004	100	W1_TWelch_FC_Up	1.566	1.021	2.947	195
**W2_DEqMS_FC_Down**	-1.615	-3.038	-1.047	8	W2_DEqMS_FC_Up	-	-	-	
**W2_Limma_FC_Down**	-1.615	-3.038	-1.047	8	W2_Limma_FC_Up	1.100	1.094	1.106	2
**W2_MSstats_FC_Down**	-3.354	-3.354	-3.354	8	W2_MSstats_FC_U	Inf	0.000	0.000	4
**W2_TStudent_FC_Down**	-1.410	-1.810	-1.047	6	W2_TStudent_FC_Up	1.100	1.094	1.106	2
**W2_TWelch_FC_Down**	-1.670	-1.810	-1.530	2	W2_TWelch_FC_Up	-	-	-	
**W3_DEqMS_FC_Down**	-1.840	-2.251	-1.355	6	W3_DEqMS_FC_Up	1.942	1.049	3.478	34
**W3_Limma_FC_Down**	-2.342	-5.357	-1.355	7	W3_Limma_FC_Up	-2.342	-5.357	-1.355	7
**W3_MSstats_FC_Down**	All -inf	All -inf	All -inf	49	W3_MSstats_FC_U	All Inf	All Inf	All Inf	22
**W3_TStudent_FC_Down**	-	-	-	-	W3_TStudent_FC_Up	-	-	-	-
**W3_TWelch_FC_Down**	-	-	-	-	W3_TWelch_FC_Up	-	-	-	-
**W4_DEqMS_FC_Down**	-1.962	-5.656	-1.007	209	W4_DEqMS_FC_Up	1.977	1.001	11.724	260
**W4_Limma_FC_Down**	-2.048	-7.450	-1.007	228	W4_Limma_FC_Up	2.085	1.010	11.724	279
**W4_MSstats_FC_Down**	-1.888	-5.494	-1.009	222	W4_MSstats_FC_U	1.751	1.001	7.693	489
**W4_TStudent_FC_Down**	-1.991	-5.656	-1.007	186	W4_TStudent_FC_Up	2.124	1.014	11.724	207
**W4_TWelch_FC_Down**	-2.070	-5.656	-1.007	162	W4_TWelch_FC_Up	2.244	1.021	11.724	139
**W5_DEqMS_FC_Down**	-2.044	-4.368	-1.006	86	W5_DEqMS_FC_Up	2.062	1.063	6.067	57
**W5_Limma_FC_Down**	-2.257	-6.043	-1.150	76	W5_Limma_FC_Up	2.214	1.063	6.067	51

**W5_MSstats_FC_Down**	-2.773	-3.465	-2.076	47	W5_MSstats_FC_U	All Inf	All Inf	All Inf	27
**W5_TStudent_FC_Down**	-2.612	-4.368	-1.898	13	W5_TStudent_FC_Up	2.015	1.126	5.562	14
**W5_TWelch_FC_Down**	-2.920	-2.920	-2.920	1	W5_TWelch_FC_Up	2.320	1.126	5.562	6
