# Supplementary figures and images for: Reproducibility Crossroads: Impact of Statistical Choices on Proteomics Functional Enrichment
Source: Int J Mol Sci. 2025 Sep 21;26(18):9232. doi: 10.3390/ijms26189232 (PMC12471179; doi:10.3390/ijms26189232)

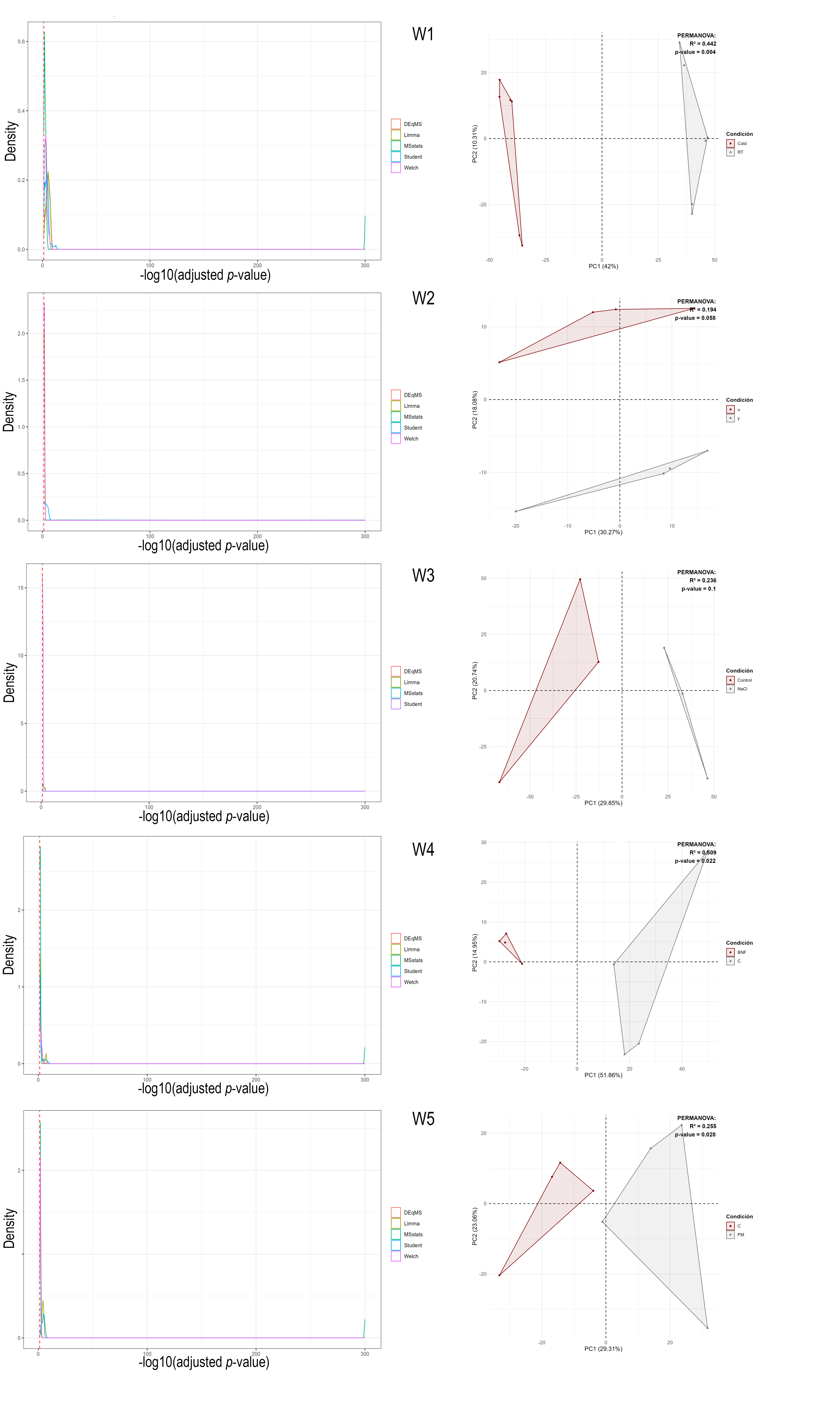

Supplement: Supplementary file 1 [file ijms-26-09232-s001.zip › SupplementaryFiles/FigureS1_15082025.tif]

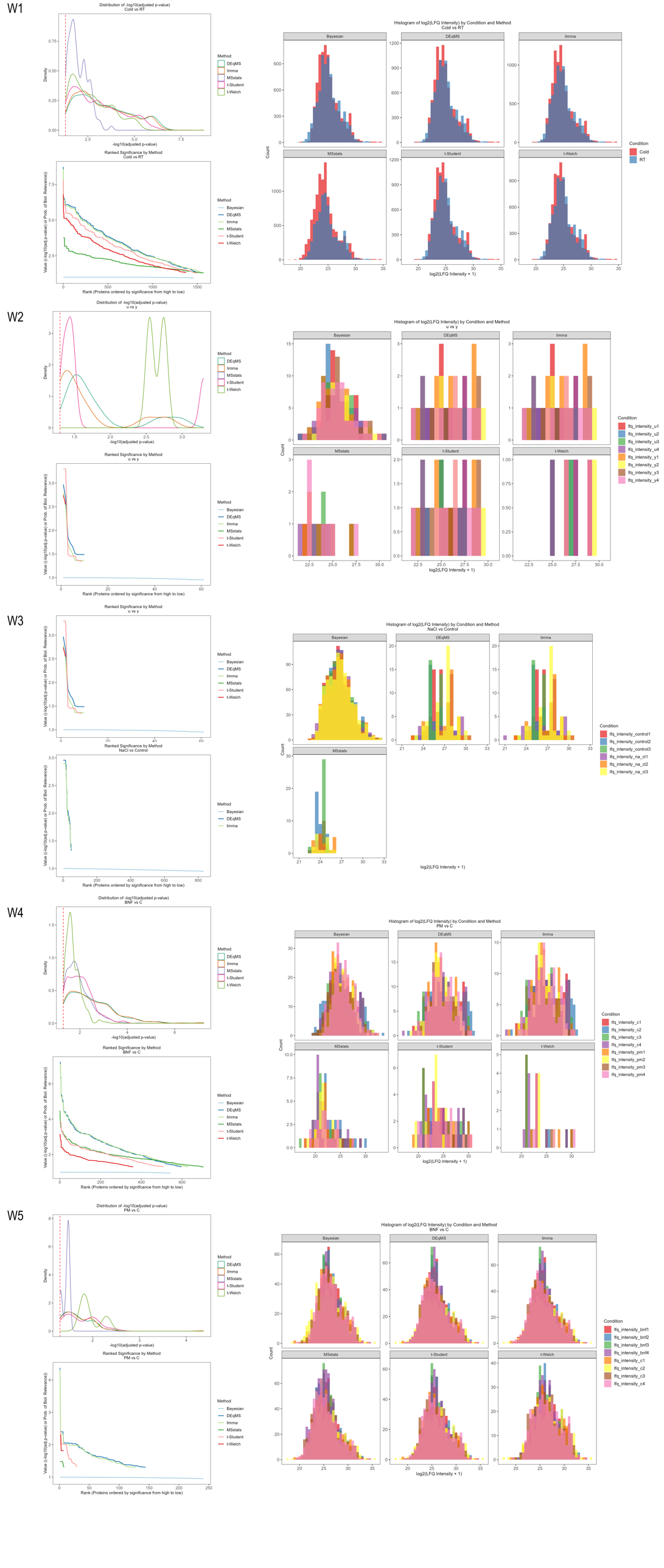

Supplement: Supplementary file 1 [file ijms-26-09232-s001.zip › SupplementaryFiles/FigureS2.tif]

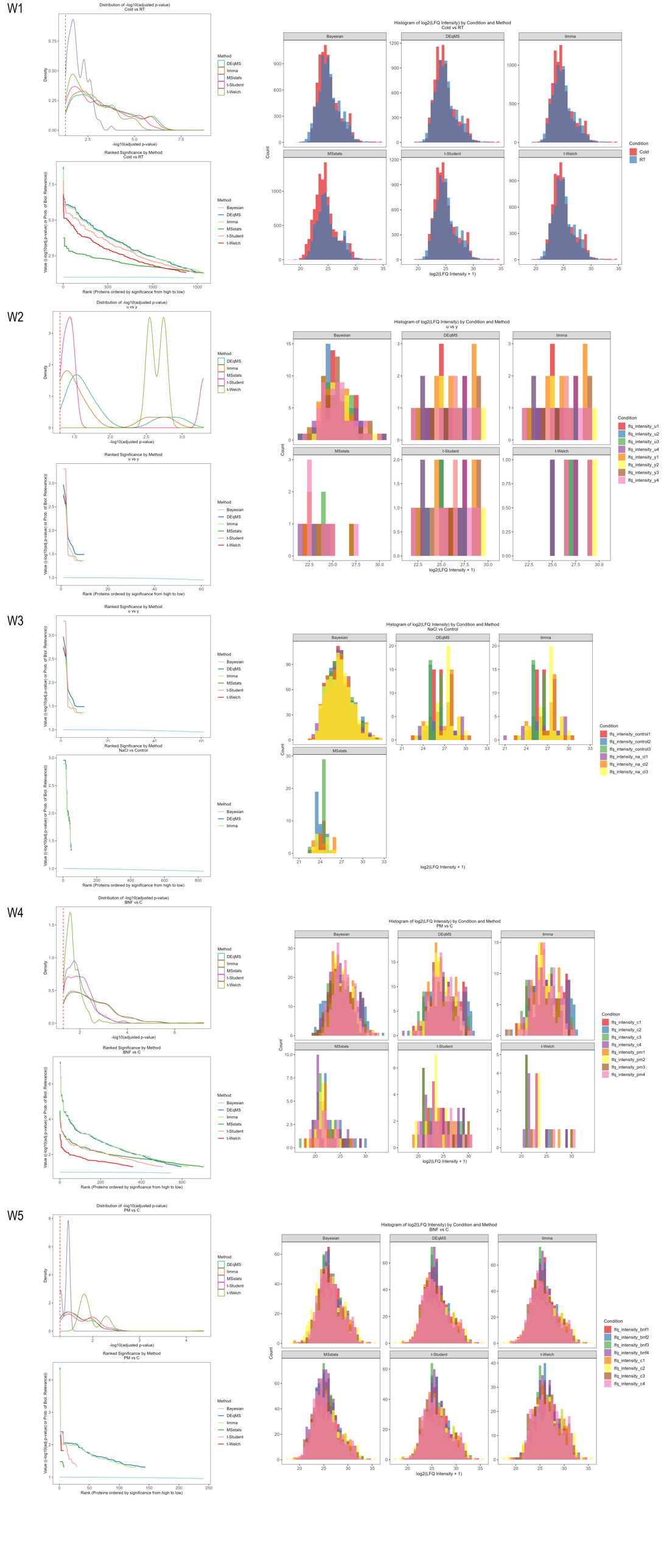

Supplement: Supplementary file 1 [file ijms-26-09232-s001.zip › SupplementaryFiles/FigureS2d.png]

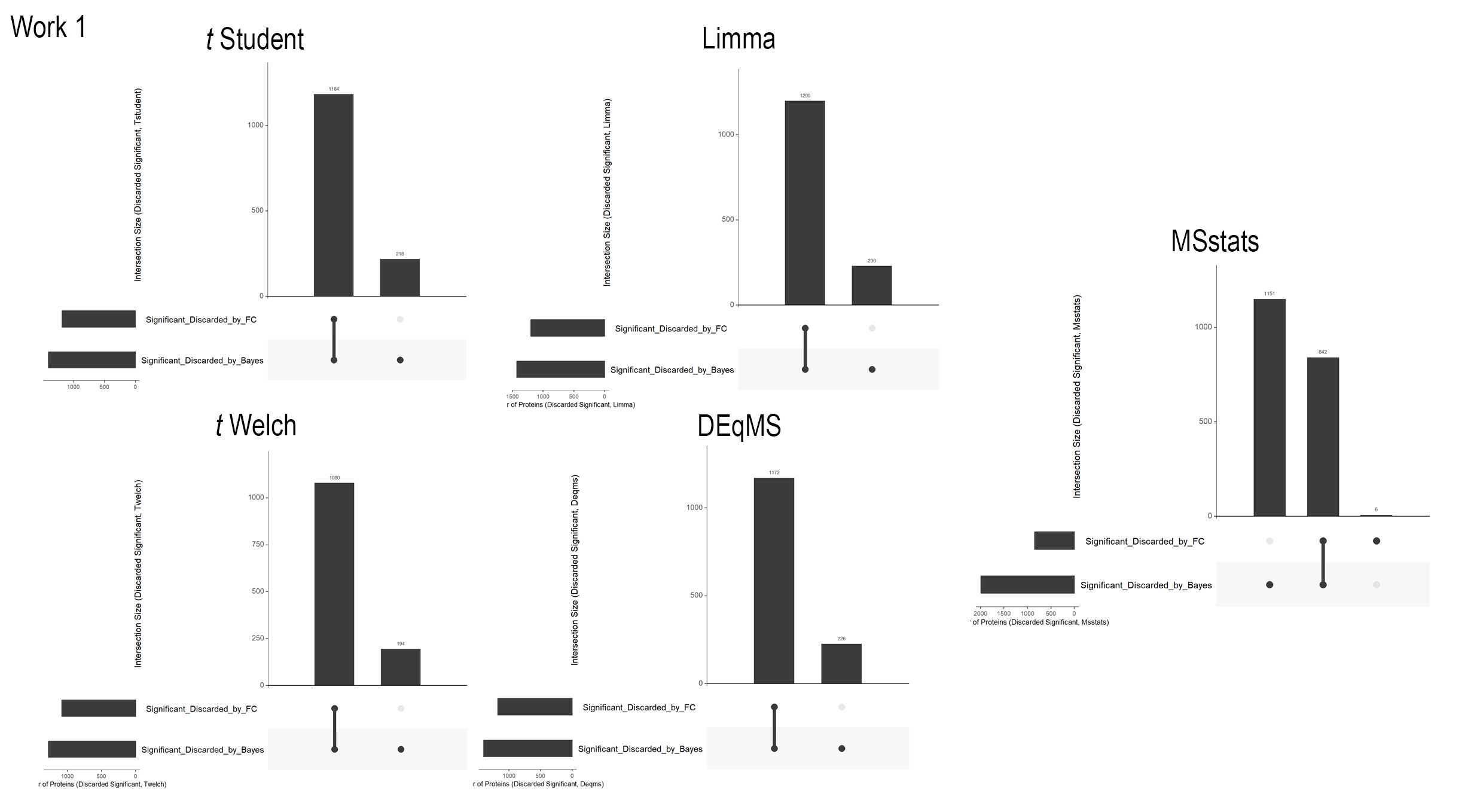

Supplement: Supplementary file 1 [file ijms-26-09232-s001.zip › SupplementaryFiles/FigureS3_03072025.tif]

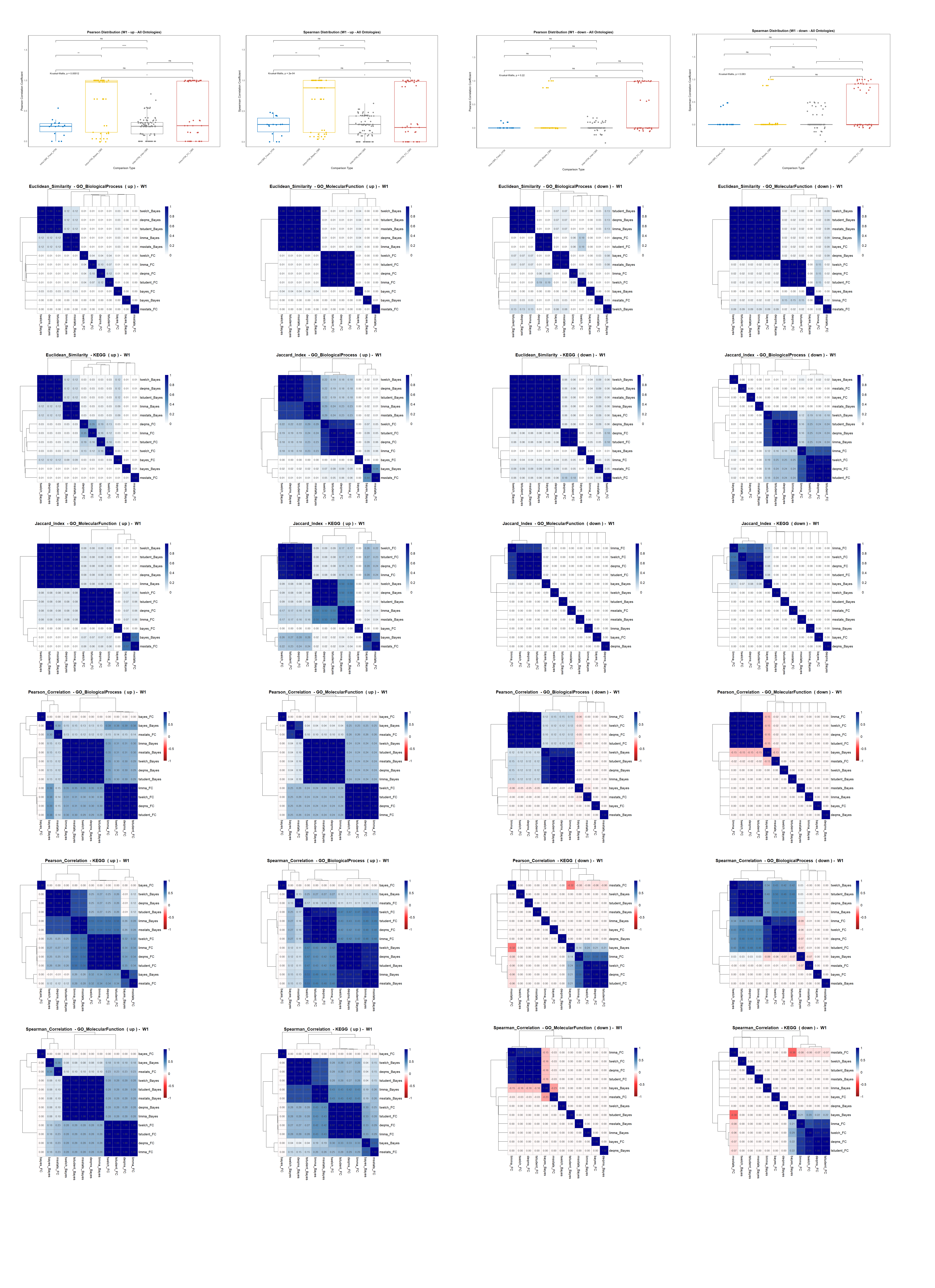

Supplement: Supplementary file 1 [file ijms-26-09232-s001.zip › SupplementaryFiles/FigureS4_06072025.tif]

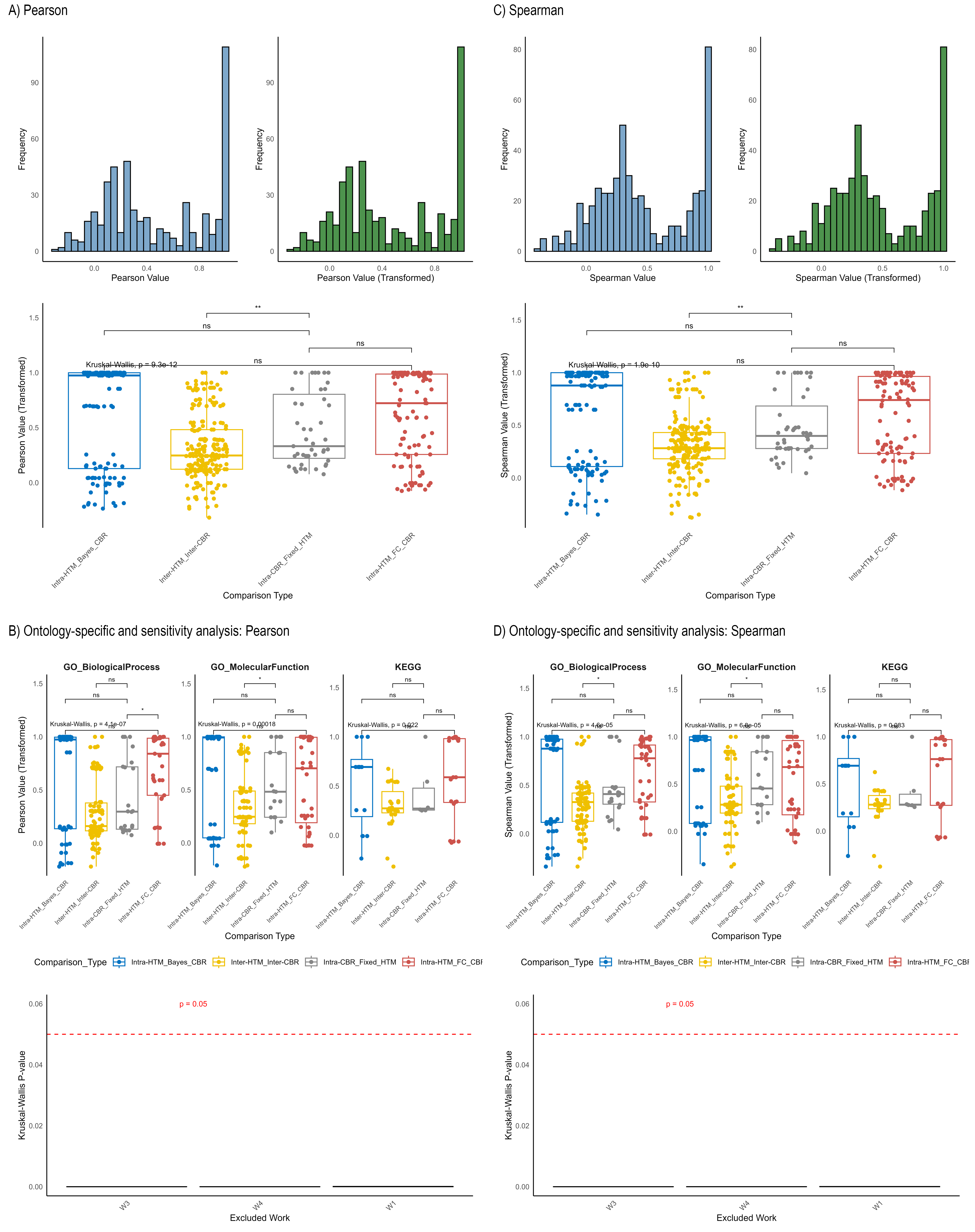

Supplement: Supplementary file 1 [file ijms-26-09232-s001.zip › SupplementaryFiles/FigureS5_06072025.tif]
